# Liver–Microbiome Crosstalk Mediates the Protective Effects of Artemisinin in *Clostridium perfringens* Models

**DOI:** 10.1111/1751-7915.70235

**Published:** 2025-10-29

**Authors:** Haodong Han, Youhao Li, Lili Wang, Zhuoya Jin, Wenqian Zhou, Bing Zhang, Can Jia, Weiqi Zhang, Yuxin Wang, Li Qiu, Song Bing, Shuhui Wang, Zhanjun Ren

**Affiliations:** ^1^ College of Animal Science and Technology Northwest A&F University Yangling Shaanxi China; ^2^ College of Veterinary Medicine Northwest A&F University Yangling Shaanxi China; ^3^ Yijun County Animal Husbandry and Technology Center Tongchuan Shaanxi China

**Keywords:** artemisinin, *Clostridium perfringens* infection, gene expression, gut microorganisms, inflammatory factors and antioxidants, mice, rabbit

## Abstract

*Clostridium perfringens* is a multi‐host opportunistic pathogen whose plasmid‐encoded toxins cause gas gangrene, necrotic enteritis and enterotoxemia, resulting in substantial economic losses in animal husbandry. In light of antibiotic bans and the need for alternatives, we employed reverse network pharmacology to screen and in vitro validate artemisinin (ART), then assessed its efficacy in murine and rabbit infection models challenged with *C. perfringens* type F. ART treatment did not significantly affect body weight change or intestinal histopathological damage. However, it significantly modulated inflammatory cytokines and antioxidant parameters in a tissue‐ and species‐dependent manner. Specifically, ART increased serum TNF‐α in mice, decreased IL‐1β in rabbits and elevated IL‐10 in multiple tissues. In addition, ART enhanced hepatic SOD and T‐AOC in mice and reduced hepatic MDA in rabbits. Microbiota analysis revealed limited and subtle shifts in community structure following ART intervention. Transcriptomic analysis further indicated that ART treatment induced marked changes in hepatic gene expression, particularly involving detoxification, lipid metabolism and stress response pathways, with notable species‐specific differences in enrichment profiles. While correlation analysis suggested associations of *Anaerotruncus* with hepatic detoxification genes and *Bacteroides* with inflammation‐regulatory genes, these genus‐level findings are based on correlation only and should be interpreted with caution given the lack of significant changes in overall microbial community structure. Collectively, these results indicate that ART can modulate host inflammatory and antioxidant responses, but its impact on gut microbiota composition in *C. perfringens* infection models appears limited, and the biological significance of observed genus‐level associations remains to be elucidated.

## Graphical abstract

Artemisinin mitigates *Clostridium perfringens* infection in mice and rabbits by modulating gut–liver crosstalk, leading to restored intestinal morphology, balanced microbiota composition and improved immunometabolic profiles.

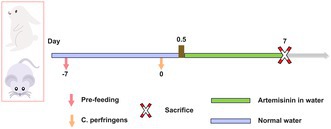

## 1 Introduction

Cases of *Clostridium perfringens* infection are common in many species, including humans (Thomas Bintsis 2017), pigs (Posthaus et al. 2020), and chickens (Moore 2016), and it relies on plasmid‐encoded toxin genes to initiate infections ranging from clostridial myonecrosis (gas gangrene) to intestinal disorders such as necrotic enteritis and enterotoxemia; based on the repertoire of these plasmid toxins, strains are classified into five toxinotypes (type A–E), each with distinct pathogenic potential (Freedman et al. 2015). Among them, the intestinal virulence of F‐type strains depends on their ability to produce *C. perfringens* enterotoxin (CPE), which causes food poisoning in humans characterised by diarrhoea, abdominal cramps, and, in severe cases, death, making these strains a common cause of foodborne illness (Shrestha et al. 2018). Animals that died from the effects of the enterotoxin exhibited pronounced intestinal bloating and distension (Ali et al. 2024).

Rabbits, with immune genes more similar to humans and susceptibility to shared pathogens (Esteves et al. 2018), are often used in intestinal immunology studies (Pasteur 1885). Conversely, although mice differ significantly from humans in their immune systems, their phylogenetic relatedness and physiological similarities make them valuable models (Perlman 2016). Therefore, rabbits and mice are commonly used as models for these diseases (Uzal et al. 2015). In particular, rabbits represent a well‐established model for investigating in vivo CPE, with histopathological features well characterised (Duncan and Strong 1969; McDonel and Duncan 1975). Mice have also been widely employed, in which lethal effects have been observed in the intestinal loop model (Yamamoto et al. 1979; Caserta et al. 2011). However, these models facilitate a more comprehensive understanding of the toxin's dose‐dependent effects and temporal dynamics; they rely on purified toxin, whereas we aim to investigate the natural pathogenic manifestations of the bacteria within the animal host.

Antibiotics are an effective means of treating bacterial diseases; however, their use is greatly restricted due to the indirect impact on human health caused by their indiscriminate use in livestock husbandry (Kupczyński et al. 2024). With stricter controls on antibiotic use, the incidence of diarrhoea in young animals—especially weanlings, which are highly susceptible to this bacterial infection and exhibit higher morbidity and mortality rates—has increased (Chen et al. 2021). Consequently, it is imperative to find alternatives to antibiotics to mitigate the adverse consequences of such infections.

Phytochemicals are an ideal alternative to antibiotics in livestock due to their multiple benefits, including improved nutrient conversion, protection against food spoilage, antimicrobial properties, improved palatability and gut health (Callaway et al. 2021). Artemisinin (ART) is one of the active ingredients extracted from *Artemisia annua* (Klayman 1985). It and its derivatives have numerous medicinal values, including antibacterial (Khan et al. 2022), antiparasitic (Solano‐Gálvez et al. 2024), anti‐inflammatory (Shi et al. 2015; Qiu et al. 2021), anticancer (Das 2015), and antioxidant properties (Liu et al. 2022). ART and its derivatives have been shown to have antibacterial activity against a wide range of pathogens. For example, it can inhibit the growth of *Helicobacter pylori* , *Staphylococcus aureus* , *Streptococcus* and *Escherichia coli* (Sisto et al. 2016; Khan et al. 2022), and the mechanism may be due to the accumulation of reactive oxygen species that it mediates (Chung et al. 2022).

It is worth noting that dysbiosis and inflammation are key features of enteritis. Given that ART has anti‐inflammatory and antibacterial properties, it may help alleviate the adverse effects of *C. perfringens* infection (CPI). Therefore, this study selected ART as a potential drug to mitigate *C. perfringens* infection based on reverse network pharmacology. We then chose mice and rabbits as models to assess whether ART could alleviate their clinical symptoms. Furthermore, we aim to explore the roles of gut microbiota and immune organs in the treatment of bacterial diseases with ART. Ultimately, we hope to identify the mechanisms through which ART exerts its effects and investigate its action pathways in different animal models.

## 2 Results

### 2.1 Artemisinin Treatment Alleviates Clinical Symptoms and Modifies Histopathological Changes in *C. perfringens* Infected Mice and Rabbits

**FIGURE 1:**
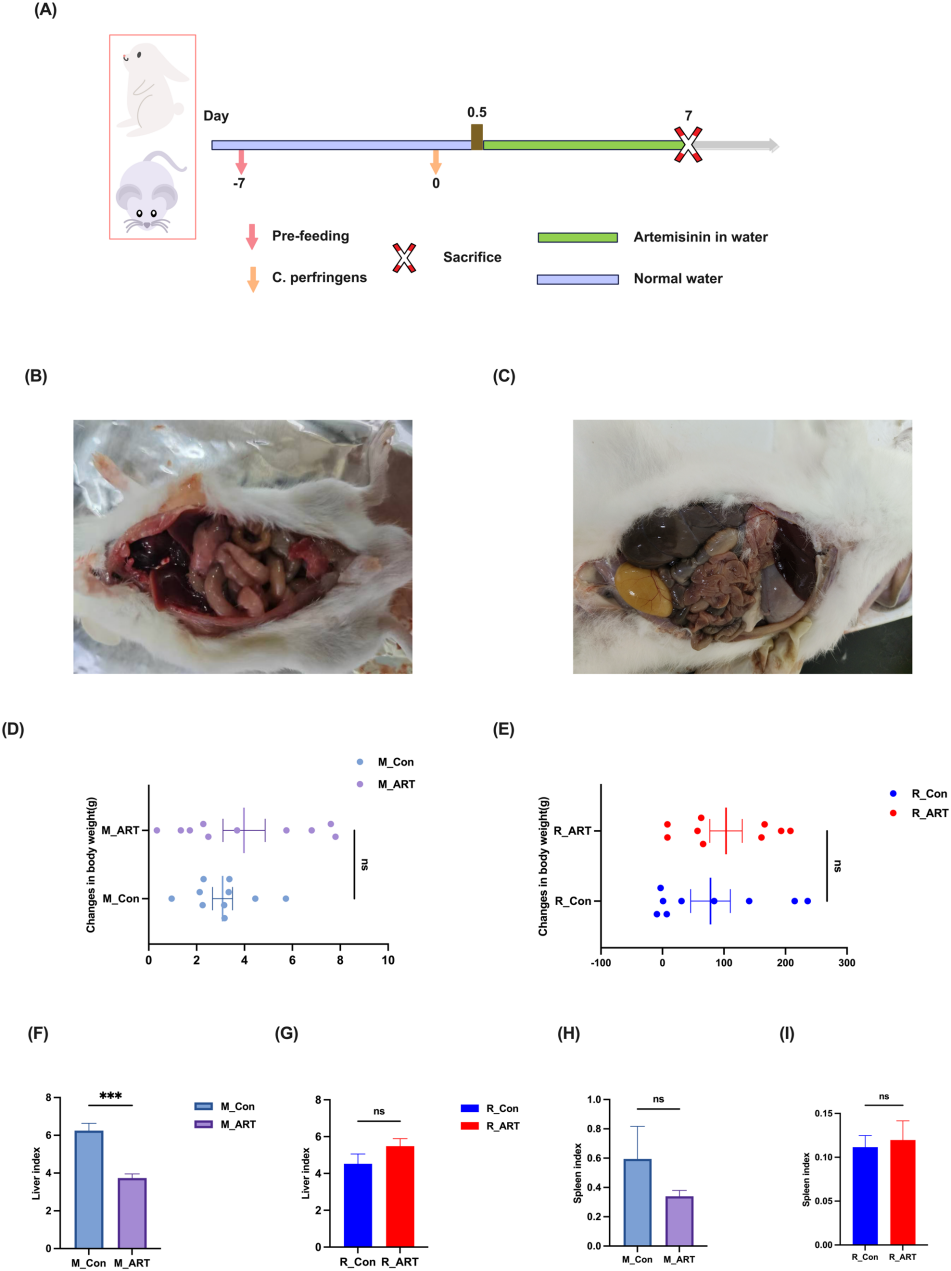
ART treatment ameliorates *Clostridium perfringens* infection in mice and rabbits. Artemisinin alleviates clinical symptoms caused by *C. perfringens* infection. (A) Timeline of animal experiments. (B, C) Intestinal gross anatomy of mice and rabbits post‐infection with *C. perfringens* . (D, E) Body weight changes in mice and rabbits over 7 days post‐infection. (F, G) Liver index in mice and rabbits after euthanasia. (H, I) Spleen index in mice and rabbits after euthanasia. *n* = 10; ***p < 0.001.

As shown in (Figure 1B,C), the intestines—especially the colon—of both mice and rabbits exhibited noticeable bloating and distension, indicating successful infection by *C. perfringens* . ART treatment did not produce a significant change in body weight; however, both murine and lagomorph models showed upward trends in weight gain at 7 days post‐infection compared to infected controls (Figure 1D,E). Strikingly, the intervention induced an extremely significant reduction in the murine hepatic index (*p* < 0.001) (Figure 1F), while its effects on lagomorph liver and spleen indices remained below detectable significance thresholds (*p* > 0.05) (Figure 1G–I).

**FIGURE 2:**
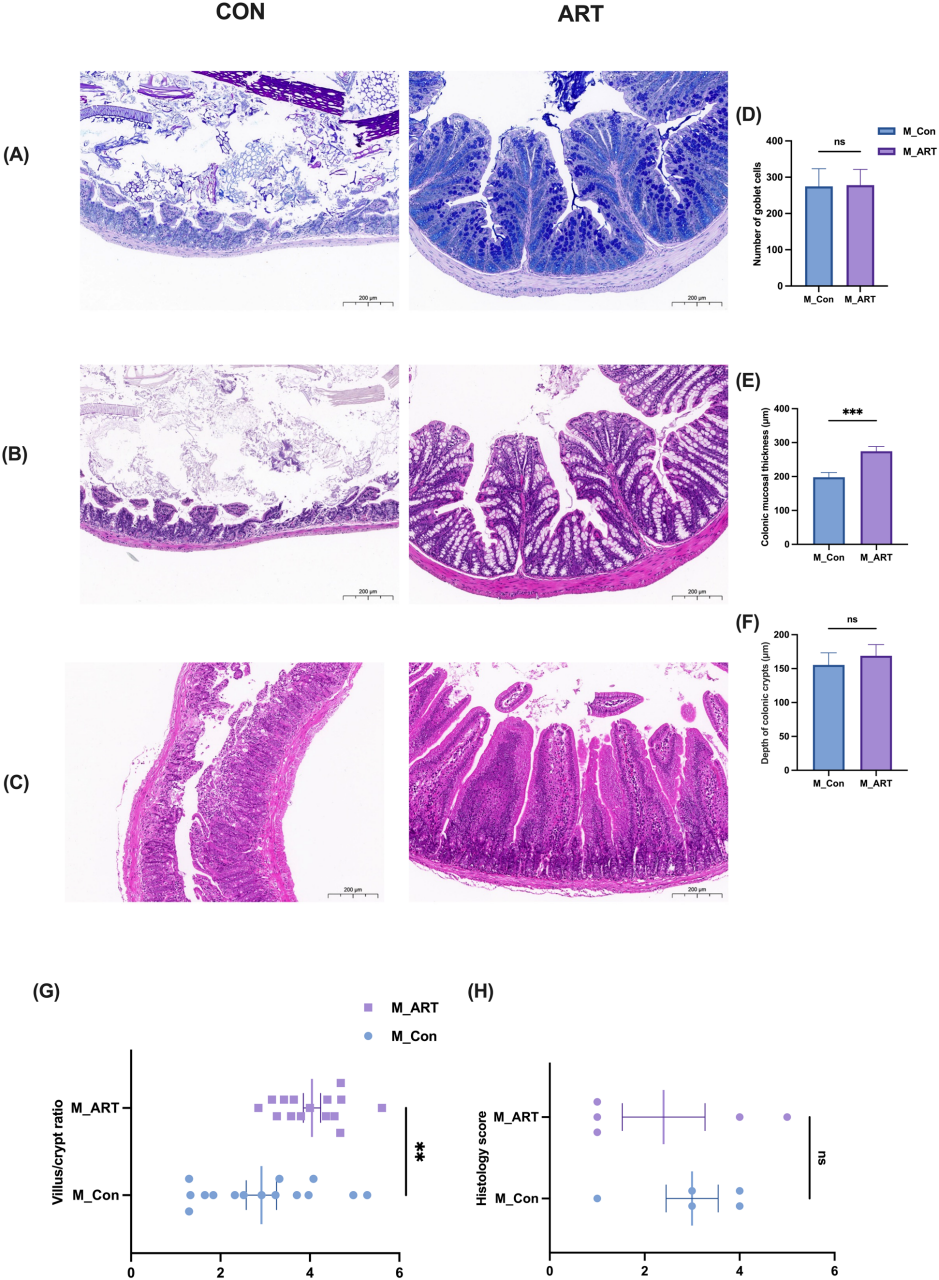
ART ameliorates intestinal structural damage induced by *Clostridium perfringens* in mice. (A) AB‐PAS staining of colon tissue. (B) H&E staining of colon tissue. (C) H&E staining of jejunum tissue. (D) Goblet cell count in colon. (E) Mucosal thickness of colon. (F) Crypt depth of colonic glands. (G) Villus height to crypt depth ratio in jejunum. (H) Histological injury score (HIS) of jejunum. *n* = 5; **p < 0.01 and ***p < 0.001.

ART demonstrated segment‐specific intestinal protection in *C. perfringens* ‐infected mice. In the jejunum, ART treatment significantly increased the villus/crypt ratio (*p* < 0.01) (Figure 2G) and preserved epithelial integrity (Figure 2C). However, no significant improvement in histological damage was observed (*p* > 0.05) (Figure 2H). In the colon, ART induced a highly significant increase in mucosal thickness (*p* < 0.001) (Figure 2E) and preserved colonic glands (Figure 2A,B), in contrast to control pathology. The absence of significant changes in goblet cell density (*p* > 0.05) (Figure 2D) or crypt depth (*p* > 0.05) (Figure 2F) suggests that protection is mediated through structural stabilisation rather than proliferative mechanisms.

**FIGURE 3:**
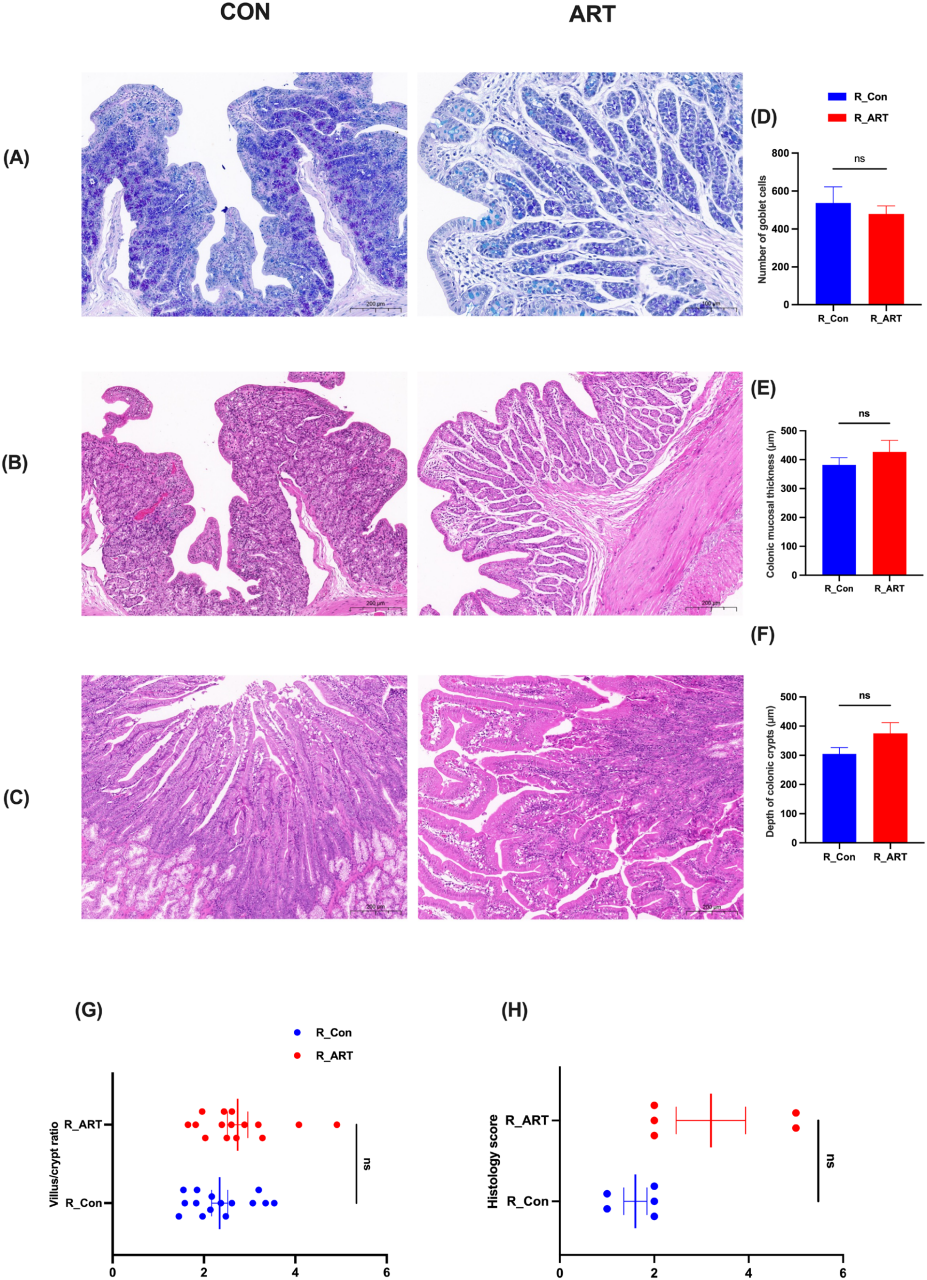
ART mitigates intestinal damage caused by *Clostridium perfringens* in rabbits. (A) AB‐PAS staining of colon tissue. (B) H&E staining of colon tissue. (C) H&E staining of jejunum tissue. (D) Goblet cell count in colon. (E) Mucosal thickness of colon. (F) Crypt depth of colonic glands. (G) Villus height to crypt depth ratio in jejunum. (H) Histological injury score (HIS) of jejunum. *n* = 5.

In rabbits, ART maintained normal colonic glandular architecture (Figure 3A,B) and duodenal villus‐Brunner's gland integrity (Figure 3C), with attenuated epithelial sloughing compared to controls. However, no significant differences were observed in colonic parameters, including goblet cell density (*p* > 0.05) (Figure 3D), mucosal thickness (*p* > 0.05) (Figure 3E) and crypt depth (*p* > 0.05) (Figure 3F), nor in duodenal measurements of villus‐crypt ratio (*p* > 0.05) (Figure 3G) and histological scores (*p* > 0.05) (Figure 3H).

### 2.2 Mice and Rabbits Infected With *C. perfringens* Exhibited Different Inflammatory and Antioxidant Responses Following Treatment With ART, With Specific Manifestations Varying According to the Species

This study measured the levels of IFN‐α, IL‐1β, IL‐6 and IL‐10 in the liver, spleen, and serum of mice and rabbits using ELISA, and assessed antioxidant indicators such as PPO, MDA, SOD, CAT and T‐AOC in the liver using colourimetric methods.

**FIGURE 4:**
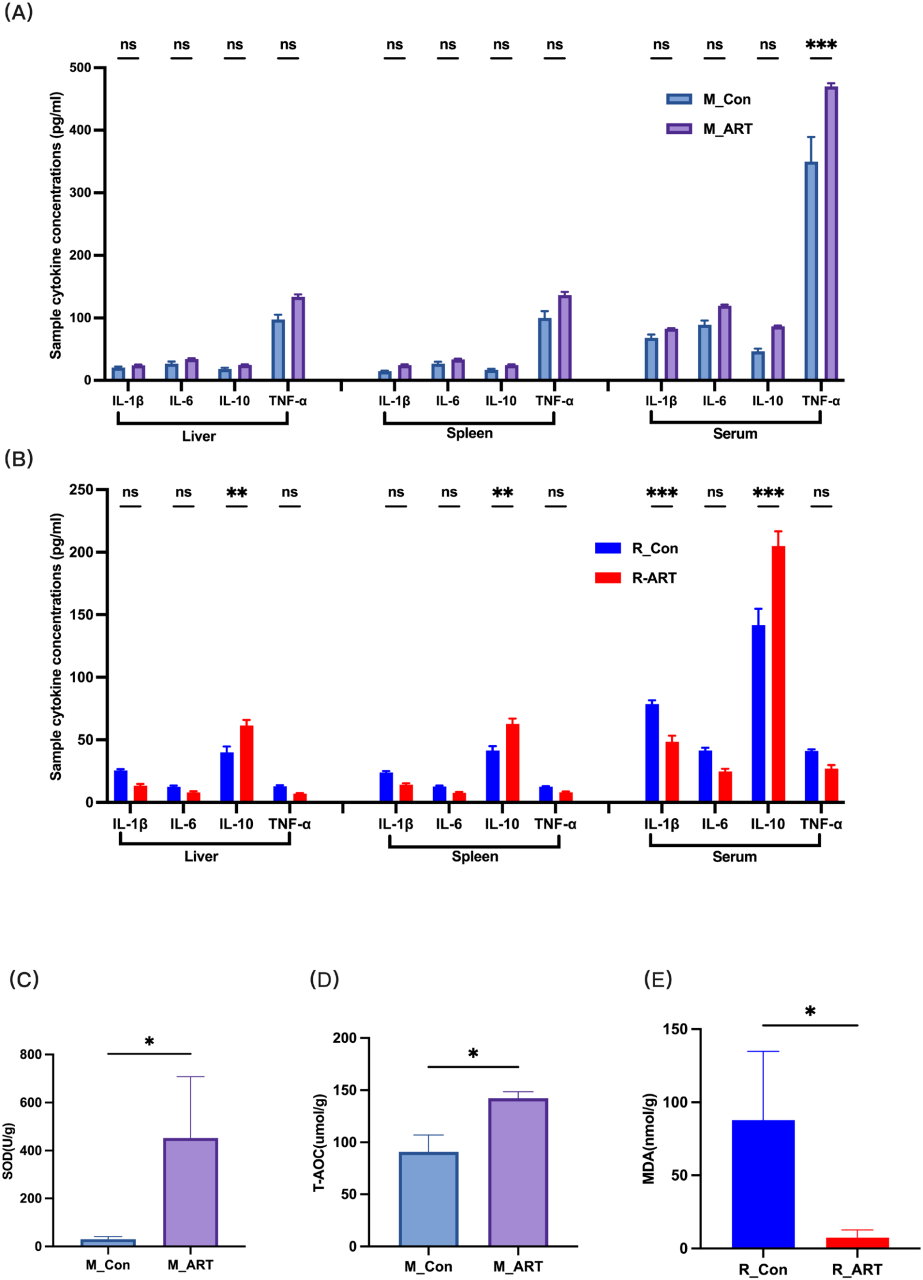
(A) The concentrations of cytokine indicators in the hepatic, splenic tissues and serum of mice. (B) The concentrations of cytokine indicators in the hepatic, splenic tissues and serum of rabbits. (C) The activity of superoxide dismutase (SOD) in the liver tissue of mice. (D) The total antioxidant capacity (T‐AOC) in the liver tissue of mice. (E) The malondialdehyde (MDA) content in the liver tissue of rabbits. *n* = 5; *p < 0.05, **p < 0.01 and ***p < 0.001.

In mice, TNF‐α levels in the ART treatment group showed an extremely significant elevation in the serum (*p* < 0.001) (Figure 4A), while no significant changes were observed in rabbits (*p* > 0.05). For IL‐1β, ART treatment led to a highly significant decrease in the serum of infected rabbits (*p* < 0.01). IL‐10 levels showed an upward trend across all tested sites, with highly significant increases in the liver and spleen (*p* < 0.01) and extremely significant increases in the serum (*p* < 0.001) (Figure 4B). No significant changes in IL‐6 were observed in either species (*p* > 0.05).

Regarding antioxidant indicators, our analysis revealed that among the parameters examined, only superoxide dismutase (SOD) and total antioxidant capacity (T‐AOC) levels in the liver of mice treated with ART exhibited significant increases compared to controls (*p* < 0.05) (Figure 4C,D). In contrast, ART significantly reduced malondialdehyde (MDA) content in the liver of rabbits (*p* < 0.05) (Figure 4E). Other antioxidant indicators are shown in Figure S1. These findings collectively suggest that the effects of ART on cytokines and the antioxidant system are highly variable across different species and tissues, highlighting the complexity of its pharmacological action.

### 2.3 Artemisinin Alleviates *C. perfringens* ‐Induced Gut Microbiota Dysbiosis in Mice and Rabbits

**FIGURE 5:**
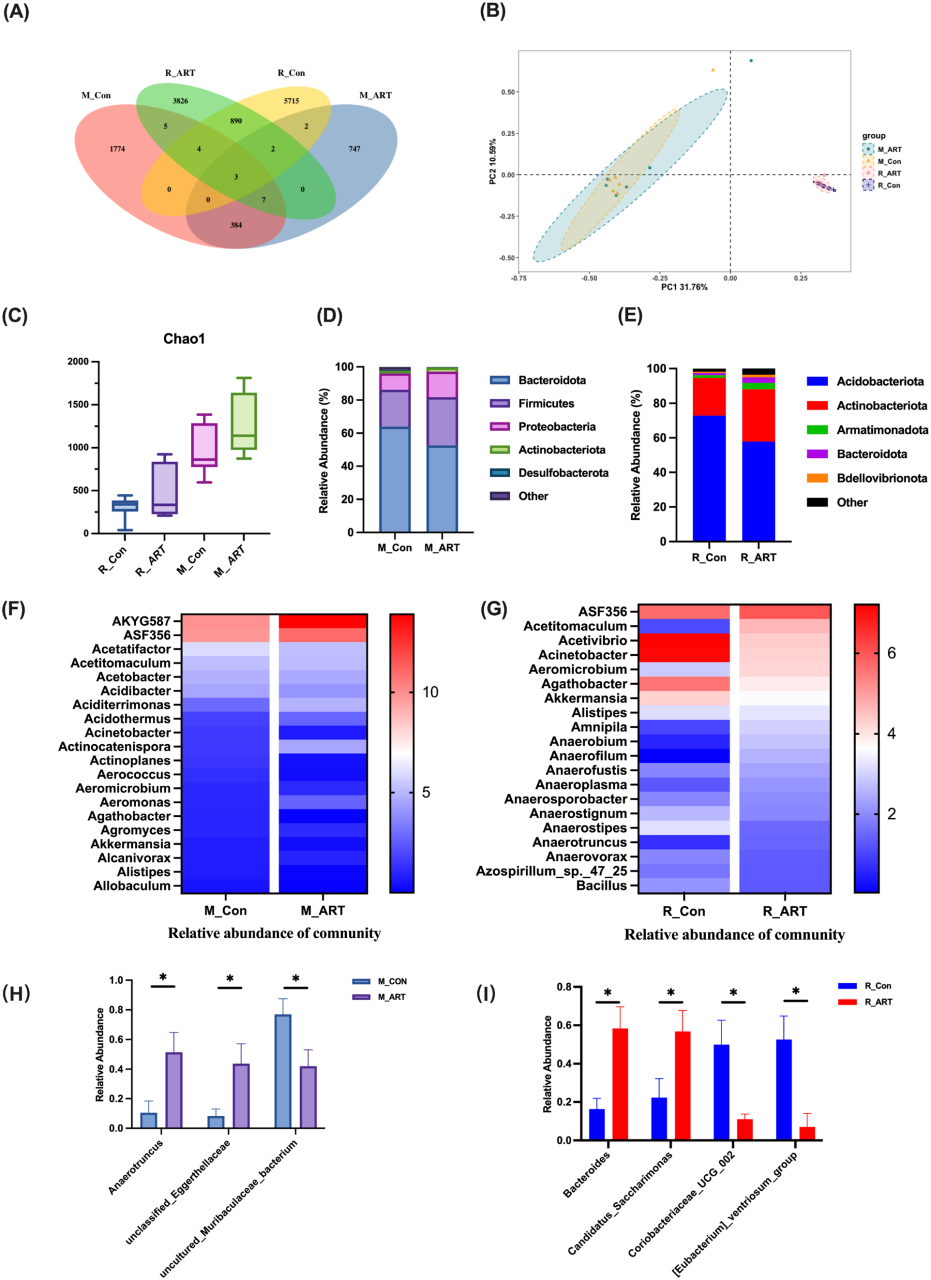
Artemisinin modulates the structure and composition of the gut microbiota in a *Clostridium perfringens* infection model. (A) The Venn diagram of M_Con, M_ART, R_Con and R_ART group. (B) PCoA score. (C) Chao index. (D) Bar chart of gut microbiota in mice at the phylum level. (E) Bar chart of gut microbiota in rabbits at the phylum level. (F) Heatmap of gut microbiota in mice at the genus level. (G) Heatmap of gut microbiota in rabbits at the genus level. (H) Relative abundance of discriminative gut microbiota in mice at the genus level. (I) Relative abundance of discriminative gut microbiota in rabbits at the genus level. Each group, *n* = 6; *p < 0.05.

In addition, principal coordinates analysis (PCoA) revealed that the samples from both control and ART‐treated groups in mice and rabbits clustered together, indicating no significant differences in overall microbial community structure between these groups. This suggests that ART administration may have only a limited or subtle impact on gut microbiota composition in *C. perfringens* ‐infected models (Figure 5B). Similarly, alpha diversity analysis showed a non‐significant trend toward increased Chao1 index in the ART groups compared to controls in both species (*p* > 0.05; Figure 5C), further supporting the notion that ART treatment did not induce substantial changes in overall microbiota diversity.

At the phylum level, a total of 32 bacterial phyla were identified in mice, with *Bacteroidota*, *Firmicutes*, *Proteobacteria*, *Actinobacteriota* and *Desulfobacteriota* being the most abundant (Figure 5D). In contrast, 17 bacterial phyla were detected in rabbits, dominated by *Acidobacteriota*, *Actinobacteriota*, *Armatimonadota*, *Bacteroidota* and *Bdellovibrionota* (Figure 5E). At the genus level, the dominant genera in both mice and rabbits exhibited only minor, non‐significant fluctuations between ART‐treated and control groups (*p* > 0.05; Figure 5F,G). Although certain genera such as *Acetitomaculum* (in rabbits) showed increased relative abundance, and others such as *Acetivibrio* and *Acinetobacter* were decreased, these changes did not reach statistical significance and may only represent trends.

Further analysis revealed that only a small number of genera showed statistically significant differences in relative abundance. For instance, *Anaerotruncus* and *unclassified_Eggerthellaceae* were increased, whereas *uncultured_Muribaculaceae_bacterium* was decreased in ART‐treated, infected mice (*p* < 0.05; Figure 5H). In rabbits, *Bacteroides* and *Candidatus_Saccharimonas* were enriched, while *Coriobacteriaceae_UCG_002* and *[Eubacterium]_ventriosum_group* were reduced following ART treatment (*p* < 0.05; Figure 5I). However, given the lack of significant shifts in overall microbial community structure, these genus‐level differences should be interpreted with caution.

Overall, ART intervention led to limited effects on gut microbiota diversity and community structure in *C. perfringens* ‐infected mice and rabbits. Although some genera exhibited statistically significant changes in relative abundance, these findings should be cautiously interpreted in the context of minimal overall community alterations.

### 2.4 Artemisinin Enhances Hepatic Metabolism to Modulate Immune Resistance

**FIGURE 6:**
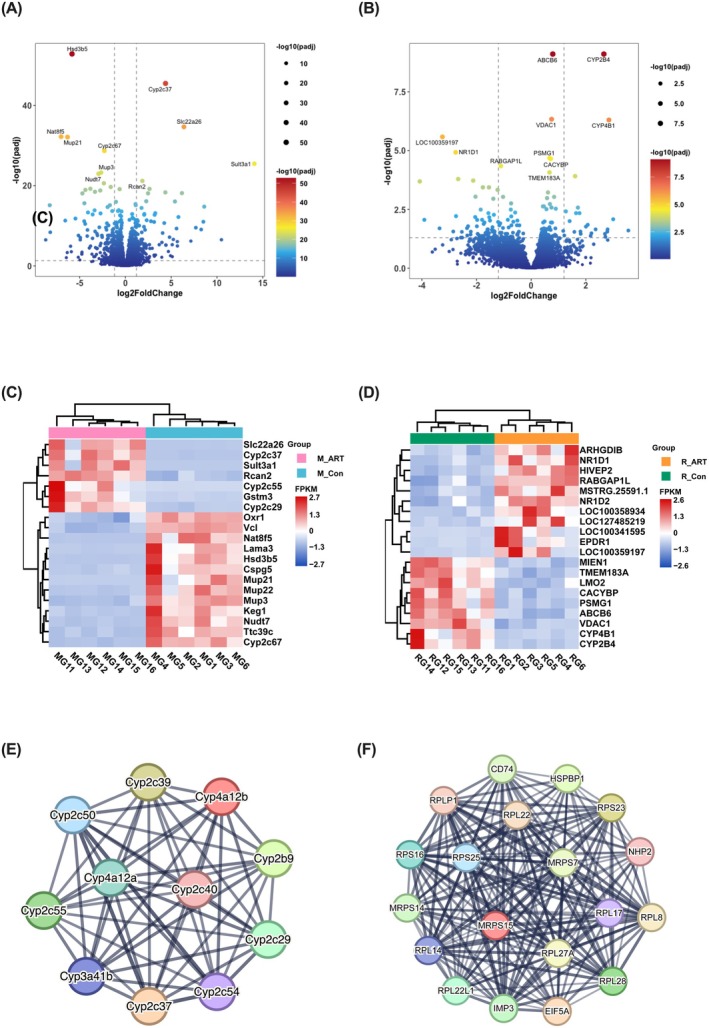
ART‐mediated remodelling of hepatic transcriptomes and PPI networks in *C. perfringens* –infected models. (A) Volcano plot analysis identifies DEGs between M_Con and M_ART groups. (B) Volcano plot analysis identifies DEGs between R_Con and R_ART groups. (C) Heat map of gene expression patterns in M_Con and M_ART groups. (D) Heat map of gene expression patterns in R_Con and R_ART groups. (E) The String software analysed the protein–protein interaction network, and the most significant module was identified in the mouse data by MCODE (score = 15.085). (F) The String software analysed the protein–protein interaction network, and the most significant module was identified in the rabbit data by MCODE (score = 21.913).

We performed transcriptome sequencing on liver tissue samples. In mice, 2648 differentially expressed genes (DEGs) were identified between the M_Con and M_ART groups (*p*_adj_ ≤ 0.05), including 237 up‐regulated and 207 down‐regulated genes (Figure 6A). In rabbits, 199 DEGs were detected under the same comparison, with 25 up‐regulated and 79 down‐regulated genes (Figure 6B). Heatmap analysis of the top 20 DEGs, ranked by *p*_adj_, revealed distinct clustering between control and ART‐treated groups in both mice and rabbits (Figure 6C,D). In mice, genes such as *Slc22a26*, *Cyp2c37*, *Sult3a1*, *Rcan2* and *Cyp2c55* were significantly up‐regulated, while *Mup3*, *Mup21*, *Mup22* and *Hsd3b5* were down‐regulated in the M_ART group (Figure 6C). In rabbits, *NR1D1*, *RABGAP1L*, *ARHGDIB* and *HIVEP2* were markedly up‐regulated following ART treatment, whereas *LMO2*, *CACYBP*, *PSMG1*, *VDAC1* and *CYP4B1* were significantly down‐regulated (Figure 6D).

For further analysis, a protein–protein interaction (PPI) network was constructed using DEGs with |log_2_FC| > 1 and *p*_adj_ < 0.05, via the STRING database (v12.0). In mice, module analysis with the MCODE plugin in Cytoscape identified a highly interconnected module consisting of 11 nodes and 50 edges, with an MCODE score of 15.085 (Figure 6E). Similarly, in rabbits, a PPI network based on the same criteria revealed a dense module composed of 19 nodes and 167 edges, with an MCODE score of 21.913 (Figure 6F).

**FIGURE 7:**
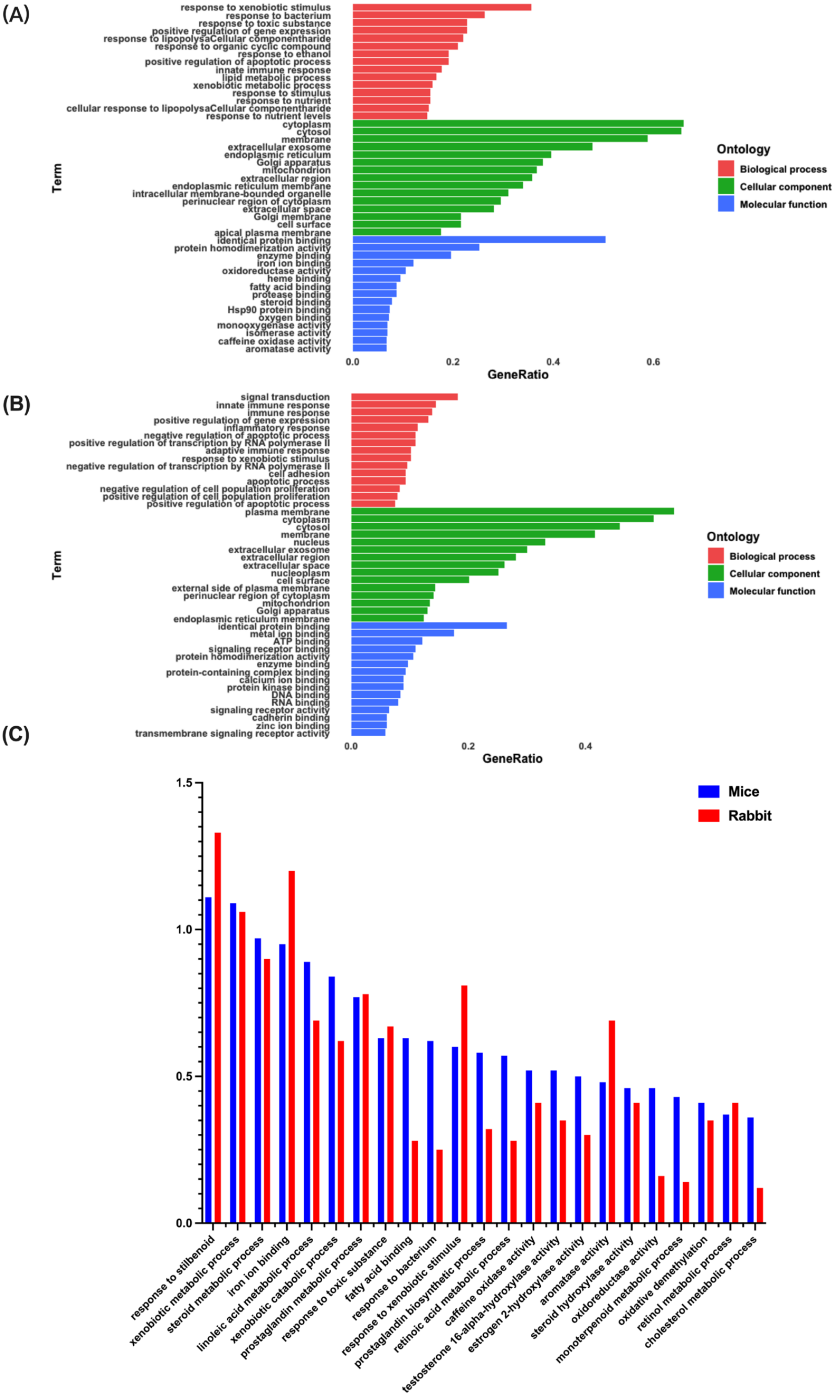
Go term enrichment analysis of DEGs reveals species‐specific and shared functions in mice and rabbits. (A) Go term enrichment analysis of DEGs in mice. (B) Go term enrichment analysis of DEGs in rabbits. (C) GO terms in the most enrichment of mice and rabbits.

GO enrichment analysis revealed that DEGs in mice were most significantly enriched in the cytoplasm, while in rabbits, the genes were enriched in the plasma membrane (Figure 7A,B). Several GO terms were commonly enriched in both species, including response to stilbenoid, xenobiotic metabolic process, steroid metabolic process, iron ion binding, linoleic acid metabolic process, xenobiotic catabolic process, prostaglandin metabolic process and response to toxic substance. These shared GO terms are primarily involved in detoxification, lipid metabolism and stress responses (Figure 7C).

**FIGURE 8:**
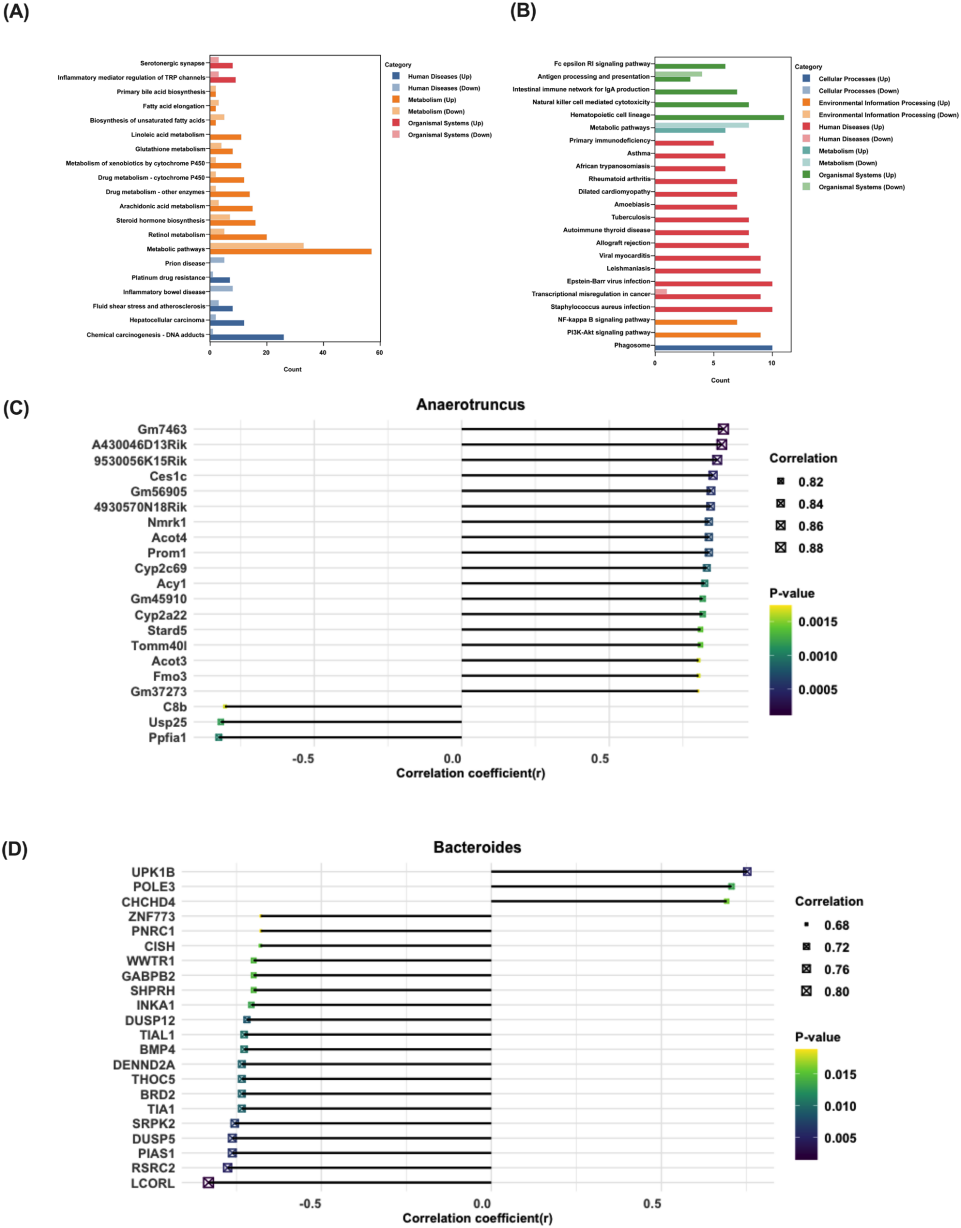
Kegg pathway analysis and Spearman's correlation of DEGs with *Anaerotruncus* and Bacteroides in mice and rabbits. (A) KEGG pathway analysis of DEGs in mice. (B) KEGG pathway analysis of DEGs in rabbits. (C) The Spearman's correlation analysis between the DEGs and *Anaerotruncus* genus by R software. (D) The Spearman's correlation analysis between the DEGs and *Bacteroides* genus by R software.

Next, KEGG enrichment analysis revealed distinct pathway profiles in mice and rabbits following ART treatment. In mice, DEGs were enriched in metabolic pathways, chemical carcinogenesis—DNA adducts, retinol metabolism, steroid hormone biosynthesis, arachidonic acid metabolism, drug metabolism—other enzymes, drug metabolism—cytochrome P450, glutathione metabolism, metabolism of xenobiotics by cytochrome P450, linoleic acid metabolism, prostaglandin metabolism, cytokine–cytokine receptor interaction, inflammatory mediator regulation of TRP channels, pathways in cancer and hepatocellular carcinoma (Figure 8A). In rabbits, DEGs were enriched in metabolic pathways, haematopoietic cell lineage, *S. aureus* infection, phagosome, transcriptional misregulation in cancer, Epstein–Barr virus infection, leishmaniasis, viral myocarditis and PI3K‐Akt signalling pathway (Figure 8B). These findings highlight distinct biological responses to ART between species.

KEGG analysis was further performed on the core genes identified by MCODE analysis from the PPI networks, which included 11 genes in mice and 19 genes in rabbits. In mice, core genes were primarily enriched in linoleic acid metabolism, retinol metabolism and arachidonic acid metabolism, while in rabbits, core genes were notably enriched in the ribosome pathway (Table 1).

**TABLE 1:** KEGG pathway enrichment analysis of core genes in mouse and rabbit (*p* < 0.05).

Term	Count%	Genes
Mouse		
Linoleic acid metabolism	17	Cyp2c29, Cyp2c55, Cyp2c39, Cyp2c37, Cyp2c54, Cyp3a41b, Cyp2c50, Cyp2c40
Retinol metabolism	12	Cyp2c29, Cyp2c55, Cyp2c39, Cyp2c37, Cyp2c54, Cyp3a41b, Cyp2c50, Cyp2b9, Cyp4a12a, Cyp4a12b, Cyp2c40
Arachidonic acid metabolism	11	Cyp2c29, Cyp2c55, Cyp2c39, Cyp2c37, Cyp2c54, Cyp2c50, Cyp2b9, Cyp4a12a, Cyp4a12b, Cyp2c40
Steroid hormone biosynthesis	10	Cyp2c29, Cyp2c55, Cyp2c39, Cyp2c37, Cyp2c54, Cyp3a41b, Cyp2c50, Cyp2b9, Cyp2c40
Chemical carcinogenesis	9	Cyp2c29, Cyp2c55, Cyp2c39, Cyp2c37, Cyp2c54, Cyp3a41b, Cyp2c50, Cyp2b9, Cyp2c40
Inflammatory mediator regulation of TRP channels	8	Cyp2c29, Cyp2c55, Cyp2c39, Cyp2c37, Cyp2c54, Cyp2c50, Cyp4a12a, Cyp4a12b, Cyp2c40
Serotonergic synapse	6	Cyp2c29, Cyp2c55, Cyp2c39, Cyp2c37, Cyp2c54, Cyp2c50, Cyp2c40
Fatty acid degradation	4	Cyp4a12a, Cyp4a12b
PPAR signalling pathway	2	Cyp4a12a, Cyp4a12b
Metabolic pathways	1	Cyp2c29, Cyp2c55, Cyp2c39, Cyp2c37, Cyp2c54, Cyp3a41b, Cyp2c50, Cyp2b9, Cyp4a12a, Cyp4a12b, Cyp2c40
Rabbit		
Ribosome	8	RPL17, RPS16, RPL27A, MRPS15, RPLP1, RPS25, RPL22, MRPS14, RPL8, RPL28, MRPS7, RPL14

### 2.5 Correlation Analysis Between Gut Microbiota and DEGs

Correlation analysis revealed that several DEGs were significantly correlated with specific bacterial genera in both mice and rabbits. In mice, genes correlated with *Anaerotruncus* abundance were mainly associated with hepatic metabolism and detoxification, including *Cyp2c69*, *Cyp2a22*, *Ces1c*, *Acot3*, *Acot4*, *Stard5*, *Fmo3* and *C8b* (Figure 8C). In rabbits, genes correlated with *Bacteroides* abundance primarily involved transcriptional regulation and cell cycle control (*ZNF773*, *BRD2*, *GABPB2*, *WWTR1* and *CISH*), RNA processing (*THOC5*, *TIA1* and *SRPK2*), signal transduction (*DUSP5* and *BMP4*) and DNA replication or repair (*POLE3* and *CHCHD4*) (Figure 8D).

## 3 Discussion

As a central immune organ, the liver senses blood‐borne pathogens and activates defence mechanisms (Gharaibeh et al. 2021). Moreover, diseases such as enteritis induce alterations in hepatic energy metabolism (Fu et al. 2022). Therefore, in enteritis driven by gut microbiota dysbiosis, research should focus not only on intestinal lesions but also on the liver's role in disease pathogenesis and therapeutic response. Using infection models in two species, we sought to elucidate the hepatic contribution to the treatment of *C. perfringens* infection and to assess the efficacy of ART as an alternative to antibiotic therapy.

*Clostridium perfringens* , an anaerobic Gram‐positive bacterium, is widely present in both environmental reservoirs and the animal gastrointestinal tract (Rajput et al. 2013). Under healthy conditions, it maintains low‐level colonisation; however, when adverse factors—such as suboptimal rearing conditions (Allaart et al. 2013), parasitic infections (Moore 2016)—disrupt the gut microbial equilibrium, *C. perfringens* can proliferate unchecked, secrete potent toxins and precipitate disease. Disruption of the intestinal epithelium by certain toxins initiates programmed cell death pathways, such as necrosis and apoptosis, leading to enteritis (Ou et al. 2024).

In the present study, the effect of ART intervention on the organism's response was systematically evaluated in a mouse and rabbit model of *C. perfringens* infection. The results showed a consistent trend of improvement in body weight gain in both animals, although the level of statistical significance was not reached. The liver is among the first organs exposed to harmful agents, including toxins and parasites, which can induce hepatic congestion and swelling (Adam et al. 2023). ART exhibits anti‐inflammatory properties (Shi et al. 2015; Qiu et al. 2021), which may account for the significant reduction in liver index observed in the ART‐treated group. As observed in our results, ART provided region‐specific intestinal protection, with more pronounced effects in mice than in rabbits. These differences suggest that both species and dosing factors may jointly influence the intestinal protective effects of ART, highlighting the need to optimise dosing strategies for different animal models in future studies.

ART modulated inflammatory cytokine profiles and hepatic antioxidant capacity in a tissue‐ and species‐dependent manner. Notably, ART increased serum TNF‐α in mice—a cytokine whose dysregulation is linked to inflammatory disease (Jang et al. 2021)—highlighting the importance of dose control. In rabbits, ART promoted an increase in IL‐10, a key anti‐inflammatory cytokine (Ouyang and O'Garra 2019), and a reduction in IL‐1β, which functions as a pro‐inflammatory factor playing a central role in inflammatory responses (van de Veerdonk et al. 2011), indicating potential effects on immune regulation and pathogen control. Regarding antioxidant markers, ART enhanced hepatic SOD and T‐AOC in mice and reduced MDA in rabbits, consistent with evidence that increased antioxidant capacity improves cellular defences and reduces oxidative damage (Kim et al. 2014). Collectively, these findings suggest that the immunomodulatory and antioxidative effects of ART may contribute to its therapeutic potential during bacterial infection, although interspecies and tissue differences highlight the need for further mechanistic investigation.

ART treatment did not result in significant changes in the overall gut microbial community structure, but some genus‐level alterations were observed. For instance, increased abundance of *Anaerotruncus* in mice, and of *Bacteroides* and *Candidatus_Saccharimonas* in rabbits, was noted. Previous studies have shown that these genera contribute to the maintenance of gut homeostasis and microbial stability. Specifically, *Anaerotruncus* abundance is positively correlated with colon length, SOD activity, and T‐AOC, and inversely correlated with inflammatory markers such as lipopolysaccharide (LPS), MDA, and myeloperoxidase (MPO) (Tang et al. 2024). *Bacteroides* can promote intestinal barrier integrity by inhibiting pathogenic colonisation and supplying nutrients to commensal microbes (Zafar and Saier Jr 2021), while *Candidatus_Saccharimonas* has been linked to the production of short‐chain fatty acids (SCFA) and increased serum IgG levels (Hong et al. 2023). Although changes in the abundance of these genera were observed, it remains unclear whether these shifts were directly attributable to experimental intervention, and their biological significance requires further investigation.

Transcriptome sequencing of liver tissue was conducted to investigate the genetic responses associated with ART intervention during *C. perfringens* infection and to explore potential links with gut microbiota. The analysis revealed DEGs in both animal models, with the extent of gene expression changes potentially influenced by species and dosing factors. Protein–protein interaction analysis indicated that genes related to drug metabolism, such as the cytochrome P450 family (Manikandan and Nagini 2018), exhibited certain changes in mice. Functional enrichment analyses (GO and KEGG) showed that these genes are primarily involved in detoxification, lipid metabolism, stress responses and immune‐related processes, with differences in pathway enrichment observed between species. These findings provide a preliminary basis for understanding the biological functions of hepatic responses to ART intervention during bacterial infection, but further investigation is needed.

Correlation analysis revealed that *Anaerotruncus* in mice was associated with the expression of multiple hepatic genes involved in metabolism and detoxification. Butyrate and other SCFAs produced by *Anaerotruncus* are proposed to enter hepatocytes via the portal vein and influence pregnane X receptor (PXR, encoded by *Nr1i2*) activity. PXR regulates cytochrome P450 enzymes and transporters (Chai et al. 2016), and, together with carboxylesterase Ces1c, drives xenobiotic metabolism (Gan et al. 2023), which could enhance hepatic clearance of ART and bacterial toxins. In rabbits, the abundance of *Bacteroides* correlated with hepatic gene expression patterns related to transcriptional regulation, cell cycle, RNA processing and signal transduction. Previous research indicates that *Bacteroides*‐derived LPS (Vatanen et al. 2016) can interact with hepatocyte TLR4 (Lu et al. 2008), activate the PI3K–Akt signalling pathway (Li et al. 2022), and subsequently influence CISH (Lv et al. 2023), a negative regulator of the JAK–STAT pathway, which is a key cascade governing inflammatory gene expression (Xin et al. 2020). The JAK–STAT pathway has been targeted for inflammatory bowel disease therapy (Nunes et al. 2017). These proposed mechanistic connections are drawn from correlation analysis only; no causal relationships have been established, and further studies are needed to clarify the specific biological significance.

In conclusion, ART treatment appeared to alleviate intestinal and hepatic disturbances in *C. perfringens* infection models, potentially through modulating inflammatory, antioxidant and microbiota profiles. While integrated analyses suggested possible liver–microbiome regulatory pathways, these associations are correlative and require further experimental investigation. Additional studies are needed to clarify the mechanisms and therapeutic potential of ART in this context.

## 4 Experimental Procedures

### 4.1 Ethics Statement

All animal experiments were approved by the Laboratory Animal Welfare and Ethics Committee of North West Agriculture and Forestry University.

### 4.2 Reverse Network Pharmacology‐Guided Screening and Iodonitrotetrazolium Chloride (INT)–Based MIC Determination for *C. perfringens*

Drug–target information related to *C. perfringens* was systematically collected by querying DrugBank (http://www.drugbank.ca/) and consulting the Clinical Application Guidelines for Antibacterial Drugs (2022 Edition) as well as the Veterinary Pharmacopoeia of the People's Republic of China (2022 Edition), supplemented by a PubMed search (2019–August 24, 2024). In total, 22 approved western drugs corresponding to 52 targets were identified. These targets were then used to query the TCMSP database, yielding 13 herbal active ingredients with explicitly predicted targets.

‘Western drug–target’ and ‘herbal ingredient–target’ networks were constructed in Cytoscape 3.8.0, and topological parameters (degree and betweenness centrality) were calculated using the NetworkAnalyzer plugin. The intersection of herb‐ and drug‐derived targets was extracted, and nodes with a degree ≥ 2× the average were defined as key targets. Herbal compounds associated with these key targets were reverse‐selected. Compound screening comprised two steps: first, Tanimoto coefficients (TS) between herbal and western drug molecules were calculated using OpenBabel, retaining pairs with TS ≥ 0.10; second, oral bioavailability (OB ≥ 30%) and drug‐likeness (DL ≥ 0.20) data from TCMSP were applied to further filter candidates, resulting in five final active ingredients.

MICs were determined following Von Mersi and Schinner (1991). Log‐phase *C. perfringens* cultures were adjusted to a 0.5 McFarland standard (≈1–2 × 10^8^ CFU/mL), diluted 1:100 in sterile saline, and inoculated into 96‐well plates containing 0.1–256 μg/mL of test compounds. After incubation at 37°C for 18–24 h, iodonitrotetrazolium chloride (INT) was added to 0.2 mg/mL and plates were incubated for an additional 30 min. Absorbance at 464 nm (OD₄₆₄) was measured, and the MIC was defined as the lowest concentration effecting ≥ 90% reduction in OD₄₆₄ compared with growth controls. The results are shown in Table S1.

### 4.3 Animals and Reagents

Six‐week‐old ICR mice and 35‐day‐old weaned Hyla rabbits were purchased from the Laboratory Animal Centre of North West Agriculture and Forestry University. All mice and rabbits were housed in the Laboratory Animal Centre of North West Agriculture and Forestry University under a 12 h light/dark cycle at an environmental temperature of 20°C ± 2°C and humidity of 45% ± 10%, with free access to sterilised standard rodent chow food and water. All animal experiments were performed by the principles outlined by the National Institutes of Health Guide for Care and Use of Laboratory Animals. The mice and rabbits were acclimatised to the new environment for at least 1 week before the start of the experiment. ART was purchased from MACLIN BioTech, with a purity of 98% (Shanghai, China).

### 4.4 CPI Model and Group

The animals were divided into four groups: M_Con (mice CPI group, *n* = 12), M_ART (mice CPI + ART group, *n* = 12), R_Con (rabbits CPI group, *n* = 12) and R_ART (rabbits CPI + ART group, *n* = 12). The F‐type *C. perfringens* used for CPI induction was isolated in our laboratory from diarrheic rabbits. CPI was induced by subcutaneous injection of 1 × 10^8^ CFU of *C. perfringens* per animal (0.1 mL for mice and 0.5 mL for rabbits).

Starting 12 h post‐injection, animals in the ART‐treated groups were administered drinking water supplemented with ART at a concentration of 0.015 mg/mL for six consecutive days. This concentration was determined based on a previous study demonstrating that a 3 mg/kg dose of the ART derivative SM934 significantly alleviated DSS‐induced colitis in mice (Yan et al. 2018). Considering the average body weight of ICR mice (~30 g) (Shin et al. 2017) and a typical daily water intake of approximately 5.8 mL per mouse (Bachmanov et al. 2002), the calculated dose corresponds well with this concentration. Although data on the use of ART and its derivatives in young rabbits remain scarce, previous studies have reported that artemether, a structurally related compound, induces reproductive toxicity and weight loss in breeding female rabbits, while doses between 1 and 3 mg/kg/day appear to be relatively safe (Clark et al. 2016). In the absence of evidence indicating adverse effects in weaned rabbits, and considering their immature physiology and potential drug sensitivity, the same concentration was applied in rabbits to ensure dosing safety and interspecies comparability. All animals were euthanised on Day 7. Body weight, faecal morphology, and clinical signs were monitored throughout the experimental period.

### 4.5 Histologic Injury Score (HIS)

Subsequent to the euthanasia of the mice and rabbits, colon tissues and duodenal samples from the rabbits, along with colon tissues and jejunal samples from the mice, were procured and subsequently fixed in a 4% paraformaldehyde solution. This was followed by embedding in paraffin. Tissue sections were stained using haematoxylin and eosin (H&E) as well as Alcian Blue‐Periodic Acid‐Schiff (AB‐PAS) staining techniques, and images were obtained through microscopy. Injuries to the duodenum and colon were assessed based on the HIS criteria (Li et al. 2020).

### 4.6 Detection of Inflammatory Cytokines and Antioxidant Indicator

After euthanasia, liver, spleen and serum samples were collected from mice and rabbits, immediately frozen at −80°C, and stored until batch analysis. Inflammatory cytokines, including interleukin‐1 beta (IL‐1β), interleukin‐6 (IL‐6), interleukin‐10 (IL‐10), and tumour necrosis factor alpha (TNF‐α), were quantified in liver, spleen and serum using commercial ELISA kits (Wuhan Jiyinmei Biotech Co. Ltd., Wuhan, China). For mice, the catalogue numbers were TNF‐α (JYM0218Mo), IL‐1β (JYM0531Mo), IL‐6 (JYM0012Mo) and IL‐10 (JYM0005Mo). For rabbits, the catalogue numbers were TNF‐α (JYM0003Rb), IL‐1β (JYM0011Rb), IL‐6 (JYM0006Rb) and IL‐10 (JYM0025Rb). Antioxidant parameters in liver samples, including polyphenol oxidase (PPO), MDA, SOD, catalase (CAT) and T‐AOC, were measured using biochemical assay kits (Beijing Biobox Biotechnology Co. Ltd., Beijing, China) via spectrophotometry, with catalogue numbers PPO (AKAO004C‐25S), MDA (AKFA013C), SOD (AKAO001C‐50S), CAT (AKAO003‐2C) and T‐AOC (AKAO012C). All assays were conducted according to the manufacturers' instructions.

### 4.7 Gut Microbiota Analysis

Microbial genomic DNA was extracted from mouse colonic and rabbit cecal contents using the DP336‐02 Soil DNA Extraction Kit (TIANGEN, Beijing, China). DNA quality and concentration were assessed on a NanoDrop 2000 (Thermo Scientific) by measuring the A260/280 ratio. The V3–V4 region of the 16S rRNA gene was PCR–amplified with primers 341F (5′‐CCTACGGGNGGCWGCAG‐3′) and 806R (5′‐GACTACHVGGGTATCTAATCC‐3′) on an ABI GeneAmp 9700 thermocycler (ABI, CA, USA) under the following conditions: initial denaturation at 95°C for 3 min; 25 cycles of 95°C for 30 s, 55°C for 30 s, 72°C for 45 s; and a final extension at 72°C for 5 min. Amplicons were purified with the AxyPrep DNA Gel Extraction Kit (Axygen Biosciences) and quantified using a Quantus Fluorometer (Promega). Libraries were prepared with the Illumina MiSeq Reagent Kit v3 and sequenced (2 × 300 bp) on an Illumina MiSeq platform. Sequence data were processed in QIIME2 (v2021.4), using DADA2 for denoising, SILVA v138 for taxonomic classification, and 97% OTU clustering. Downstream analysis included α‐ and β‐diversity metrics, taxonomic profiling and statistical comparisons.

### 4.8 Transcriptome Analysis

Total RNA was extracted from mouse and rabbit liver samples using TRIzol reagent (Invitrogen) according to the manufacturer's protocol. RNA integrity and purity were assessed on an Agilent 2100 Bioanalyzer and LabChip GX system (PerkinElmer), and concentrations were measured on a NanoDrop 2000 spectrophotometer (Thermo Scientific). For library construction, 2 μg of total RNA per sample was input into the VAHTS Universal V6 RNA‐seq Library Prep Kit for Illumina (Vazyme Biotech, Nanjing, China). Libraries were sequenced on an Illumina NovaSeq X Plus platform (2 × 150 bp reads).

Raw reads were trimmed for adapters and low‐quality bases using Trimmomatic, then aligned to the reference genomes (mm10 for mouse, OryCun2.0 for rabbit) with HISAT2. Transcript assembly and quantification were performed with StringTie, and gene expression levels were normalised to FPKM. Genes with FPKM ≥ 0.1 in at least one sample were considered expressed. Differential expression analysis between groups was carried out using DESeq2 (v1.46.0) with thresholds of adjusted *p* < 0.05 and |log_2_ fold‐change| ≥ 1.

GO enrichment was performed using the GOseq package (accounting for gene length bias), and KEGG pathway enrichment was assessed via KOBAS. Enrichment significance was evaluated by rich factor (ratio of DEGs to total annotated genes), count of DEGs in each pathway, and *q* < 0.05 after multiple testing correction.

### 4.9 Quantitative Real‐Time PCR

Total RNA from liver tissue was extracted using TRIzol (Vazyme). Reverse transcription, amplification and relative quantification of mRNA (2^−ΔΔCT^ method) were performed in triplicate on a CFX Opus 96 Real‐Time PCR System (Bio‐Rad) using the TB Green Premix Ex Taq II kit, with B2m and 18S rRNA serving as internal reference genes for mice and rabbits, respectively. Primer and results sequences are listed in Table S2 and Figure S2.

### 4.10 PPI Network

PPI network of DEGs was constructed by Search Tool for the Retrieval of Interacting Genes (STRING; https://string‐db.org). Then the PPIs of these DEGs were visualised in Cytoscape software (Cytoscape, 3.7.2), and significant modules in the PPI network were identified by molecular complex detection (MCODE).

### 4.11 Correlation Analysis

Correlation analysis between the DEGs and differential genera in mice and rabbits was performed separately using the Spearman correlation coefficient in R. The results were then visualised in R, with the most significant DEGs selected for visualisation, and hub genes were identified based on a *p*‐value < 0.05.

### 4.12 Statistical Analysis

The data were expressed as means ± standard error of the mean (SEM). A t‐test was performed for comparison between two groups by R. *p* < 0.05 (*), *p* < 0.01 (**) and *p* < 0.001 (***) were considered as statistical significance.

## Author Contributions

**Haodong Han:** conceptualization, methodology, writing – original draft, formal analysis. **Youhao Li:** investigation, writing – review and editing, data curation. **Lili Wang:** investigation, visualization. **Zhuoya Jin:** investigation, visualization. **Wenqian Zhou:** resources, software, visualization. **Bing Zhang:** software, resources, visualization. **Can Jia:** visualization, resources, software. **Weiqi Zhang:** software, resources, visualization. **Yuxin Wang:** visualization, software, resources. **Li Qiu:** software, resources, visualization. **Song Bing:** resources. **Shuhui Wang:** resources. **Zhanjun Ren:** writing – review and editing, project administration, supervision, funding acquisition.

## Conflicts of Interest

The authors declare no conflicts of interest.

## Supporting information


**Table S1:** Screening and evaluation of candidate herbal compounds targeting *Clostridium perfringens* .
**Table S2:** Primers for RT‐qPCR analysis.
**Figure S1:** Antioxidant indicators in rabbits and mice infected with *Clostridium perfringens* with or without artemisinin treatment. *n* = 5; *p < 0.05, **p < 0.01 and ***p < 0.001.
**Figure S2:** mRNA expression of genes in the liver tissue. Real‐time PCR analysis for the mRNA expression of relevant genes was performed on the liver tissue; values are presented as the mean + standard of the mean (SEM). *n* = 5 for each treatment. *p < 0.05, **p < 0.01 and ***p < 0.001.

## Data Availability

The data that support the findings of this study are available from the corresponding author upon reasonable request.
