# Supplementary material for: Liver–Microbiome Crosstalk Mediates the Protective Effects of Artemisinin in Clostridium perfringens Models
Source: Microb Biotechnol. 2025 Oct 29;18(11):e70235. doi: 10.1111/1751-7915.70235 (PMC12569453; doi:10.1111/1751-7915.70235)
Supplement: Supplementary file 1 — Table S1: Screening and evaluation of candidate herbal compounds targeting Clostridium perfringens . Table S2: Primers for RT‐qPCR analysis. Figure S1: Antioxidant indicators in rabbits and mice infected with Clostridium perfringens with or without artemisinin treatment. n = 5; *p < 0.05, **p < 0.01 and ***p < 0.001. Figure S2: mRNA expression of genes in the liver tissue. Real‐time PCR analysis for the mRNA expression of relevant genes was performed on the liver tissue; values are presented as the mean + standard of the mean (SEM). n = 5 for each treatment. *p < 0.05, **p < 0.01 and ***p < 0.001. [file MBT2-18-e70235-s001.docx]

Application of Reverse Network Pharmacology for Screening Herbal Compounds Targeting *Clostridium perfringens*

A total of 22 drugs were identified as being associated with Clostridium perfringens, corresponding to 52 unique targets.
Representative drugs and their associated target counts include: penicillin G (4), metronidazole (3), bacitracin (3), clindamycin (1), vancomycin (1), ceftriaxone (5), erythromycin (3), doxycycline (1), chloramphenicol (3), fidaxomicin (1), quinacrine (4), amoxicillin (1), meropenem (1), tetracycline (8), and tylosin (0).
By querying the TCMSP database with these 52 targets, 13 herbal active ingredients with clearly predicted target interactions were retrieved.
Using Tanimoto similarity calculations (TS ≥ 0.10), the following compound pairs were identified:
TS > 0.10:
- Hyperforin–Rifampin: 0.12
- Diosgenin–Rifaximin: 0.11
- Diosgenin–Rifampin: 0.11
- Artemisinin–Rifampin: 0.11
- Progesterone–Phytonadione: 0.11
TS = 0.10:
- Tanshinone IIA–Rifaximin
- Tanshinone IIA–Rifampin
- Kaempferol–Rifaximin
- Hyperforin–Rifaximin
- Quercetin–Rifaximin
- Streptomycin C–Rifampin
- Doxycycline–Streptomycin C
Following ADME screening (OB ≥ 30%, DL ≥ 0.20), seven herbal ingredients were retained:
- Kaempferol (OB: 0.4188, DL: 0.24)
- Hyperforin (OB: 0.4403, DL: 0.60)
- Diosgenin (OB: 0.8088, DL: 0.81)
- Coumestrol (OB: 0.3249, DL: 0.34)
- Artemisinin (OB: 0.4988, DL: 0.31)
- Tanshinone IIA (OB: 0.4989, DL: 0.40)
- Quercetin (OB: 0.4643, DL: 0.28)
Integrating both Tanimoto similarity and ADME properties, the following seven candidate herbal ingredients were prioritized:
Quercetin, Hyperforin, Diosgenin, Coumestrol, Artemisinin, Tanshinone IIA, and Kaempferol.
Based on clinical relevance and cost considerations, five compounds were selected for experimental validation:
Quercetin, Diosgenin, Artemisinin, Tanshinone IIA, and Kaempferol.
The effective concentrations (mg/mL) determined for each compound were as follows:
- Artemisinin: 0.006835938
- Tanshinone IIA: 0.01171875
- Diosgenin: 0.04296875
- Kaempferol: 0.01953125
- Quercetin: 0.0234375
Preliminary results indicate that Artemisinin exhibits the strongest inhibitory effect against Clostridium perfringens, as evidenced by the lowest effective concentration among the tested compounds.

Table S1. Screening and evaluation of candidate herbal compounds targeting Clostridium perfringens

| Compound Name | Tanimoto Similarity (TS) | OB (%) | DL | Selected for MIC Test | Effective Concentration (mg/mL) |
| --- | --- | --- | --- | --- | --- |
| Quercetin | 0.10 | 46.43 | 0.28 | Yes | 0.0234375 |
| Tanshinone IIA | 0.10 | 49.89 | 0.40 | Yes | 0.01171875 |
| Diosgenin | 0.11 | 80.88 | 0.81 | Yes | 0.04296875 |
| Artemisinin | 0.11 | 49.88 | 0.31 | Yes | 0.006835938 |
| Kaempferol | 0.10 | 41.88 | 0.24 | Yes | 0.01953125 |
| Hyperforin | 0.12 | 44.03 | 0.60 | No | — |
| Coumestrol | — | 32.49 | 0.34 | No | — |

Note: OB = Oral Bioavailability; DL = Drug-Likeness. MIC evaluation was performed using INT (iodonitrotetrazolium chloride) colorimetric assay; effective concentration was defined as the lowest concentration resulting in ≥90% OD₄₆₄ reduction compared to growth control.

Artemisinin demonstrated the most potent inhibitory effect.Supplementary Results

Antioxidant indicators


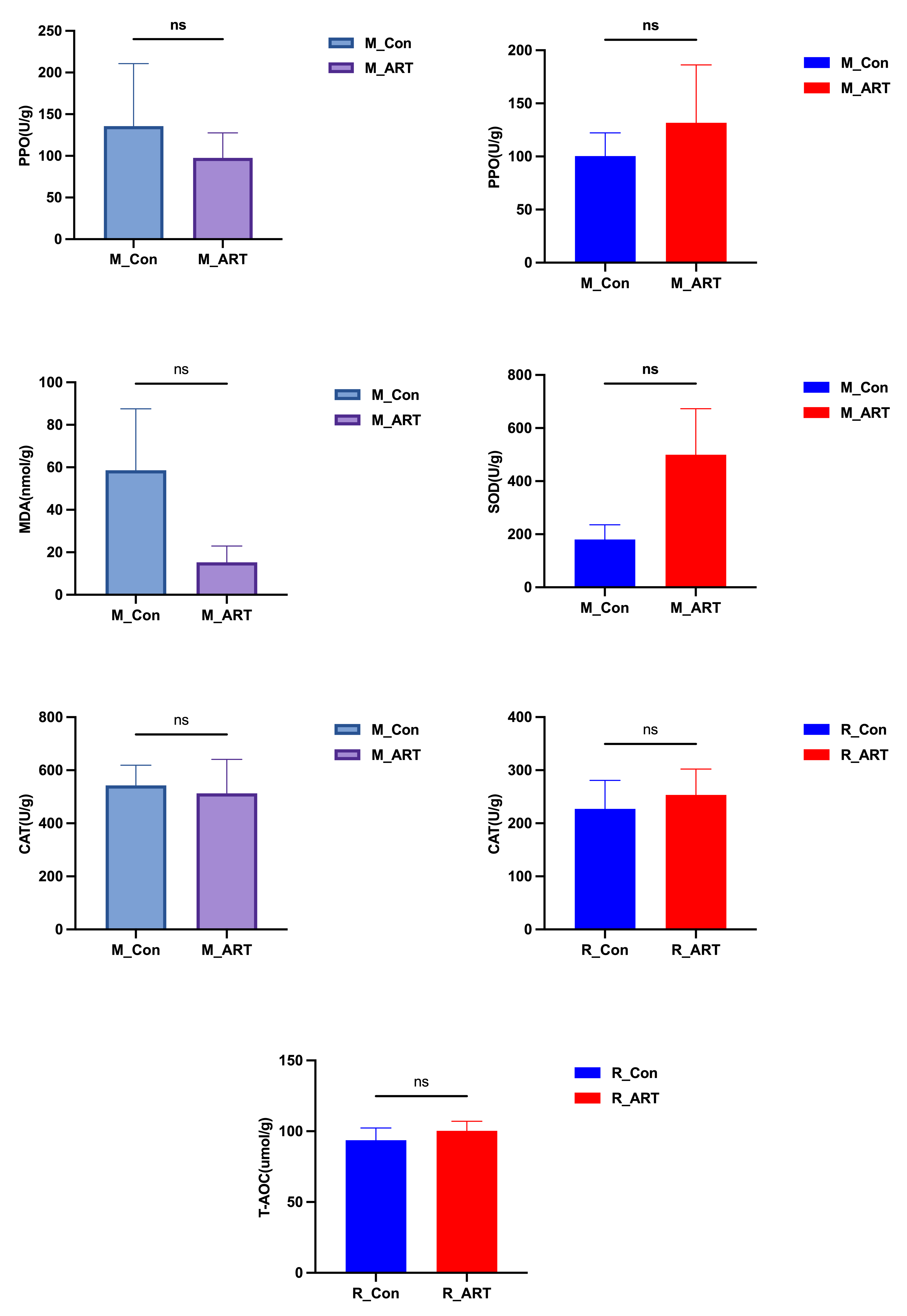


Fig S1. Antioxidant indicators in rabbits and mice infected with *Clostridium perfringens* with or without artemisinin treatment. n=5; **P* < 0.05, ***P* < 0.01, ****P* < 0.001.

Validation of transcriptome sequencing results by RT-qPCR

Table S2. Primers for RT-qPCR analysis

|  | Forward primer | Reverse primer |
| --- | --- | --- |
| 18S rRNA | GACACGGACAGGATTGAC | TCGCTCCACCAACTAAGA |
| B2m | CAGCAAGGACTGGTCTTTCTAT | AACTCTGCAGGCGTATGTATC |
| Cyp2c29 | CACAGCTAAAGTCCAGGAAGAG | GAATCATGGCGTCTGTATAGGG |
| Cyp2c37 | GGTTCACCCAAAGGACATAGA | TCAGGAAGGAATGAAGCAGAG |
| Cyp2c55 | GGACTCTTGCTCCTACTGAAAC | ATGTACCATGGCATCTGTGTAG |
| CYP4B1 | TACGCCCTCCATAGGAACA | GAGAAGGGAATGAAGGCATAGG |
| NR1D1 | CTTCAATGCCAACCACACATC | CATTCAGGGCCTCGTTATGA |
| RABGAP1L | GTCTATGGTCCCAACCAAGAAG | TATCTGTCCATCGGGTCCTC |


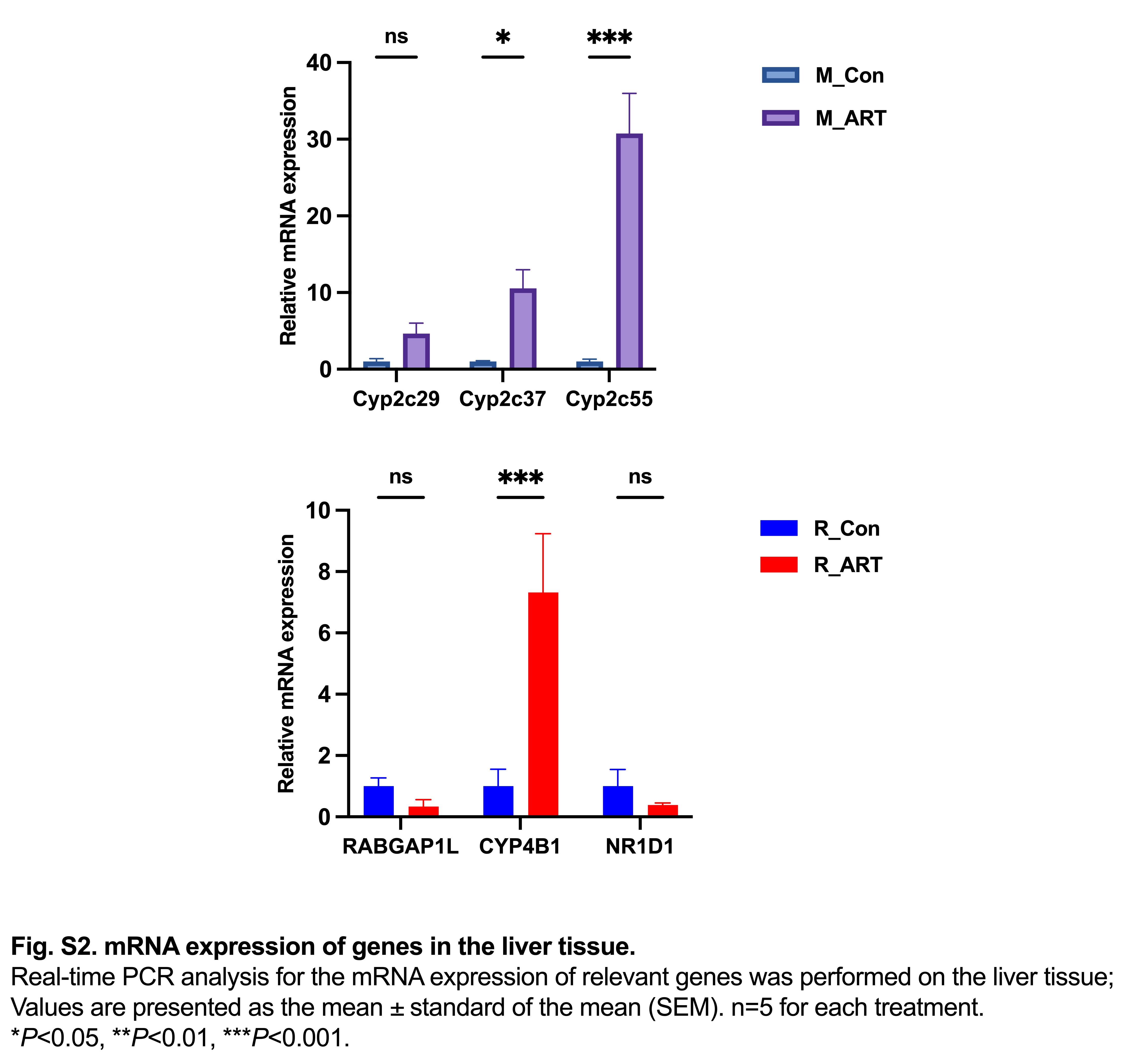


Fig S2. mRNA expression of genes in the liver tissue.

Real-time PCR analysis for the mRNA expression of relevant genes was performed on the liver tissue; Values are presented as the mean + standard of the mean (SEM). n=5 for each treatment. **p*<0.05, ***p*<0.01, ****p*<0.001.
